# Metabolic syndrome: is equine disease comparable to what we know in humans?

**DOI:** 10.1530/EC-14-0038

**Published:** 2014-06-24

**Authors:** Antonia Ertelt, Ann-Kristin Barton, Robert R Schmitz, Heidrun Gehlen

**Affiliations:** Equine Clinic, Free University of Berlin Berlin Germany

**Keywords:** metabolism, obesity, diabetes, cardiovascular, inflammation

## Abstract

This review summarizes similarities and differences between the metabolic syndromes in humans and equines, concerning the anatomy, symptoms, and pathophysiological mechanisms. In particular, it discusses the structure and distribution of adipose tissue and its specific metabolic pathways. Furthermore, this article provides insights and focuses on issues concerning laminitis in horses and cardiovascular diseases in humans, as well as their overlap.

## Introduction

Metabolic syndrome in humans was first described almost 50 years ago by Camus (1), but there was little interest in this disease until the late 1980s, when it gained new attention as ‘syndrome X’ or ‘the deadly quartet’ (2, 3).

According to the Adult Treatment Panel III of the National Cholesterol Education Program (NCEP) and the American Heart Association/National Heart, Lung and Blood Institute (http://www.nhlbi.nih.gov/health/health-topics/topics/ms/), three of the five criteria – waist circumference, hypertension, elevated glucose levels in the fasting state, elevated triglycerides, and decreased HDL cholesterol – have to be met to establish the diagnosis of human metabolic syndrome (HMS) (4).

A similar disease in horses, called ‘equine metabolic syndrome’ (EMS), was first described by Johnson *et al*. (5) and commonly accepted by a consensus committee and the veterinary public. Although equine disease resembles what we know from humans in many aspects, distinct differences have been defined concerning the vascular structures affected by the disease, typically the coronary vessels in humans, while horses present with an increased risk of laminitis (6).

As pituitary pars intermedia dysfunction (PPID, equine Cushing's syndrome), EMS is a common endocrinologic disease with severe metabolic consequences in equine medicine, and tends to affect young horses. EMS is a very complex disorder that has been the focus of many prior studies, as is HMS in human medicine (7).

In this paper, we aimed to review similarities and differences between EMS and HMS, as this may allow transfer of knowledge between both species.

## Anatomy and physiology of adipose tissue

Adipose or fat tissue, a special form of reticular connective tissue, consists mainly of adipocytes. White and brown adipose tissues in both horses and humans must be differentiated: the former tissue contains single lipid droplets and the latter contains numerous smaller droplets. In addition to serving as fat storage, adipose tissue has endocrine functions (8, 9, 10).

When weight is gained physiologically, the number of fat cells does not change, but more lipids are stored in fat cells, thereby causing an increase in cell size. By contrast, obesity is characterized not only by hypertrophic expansion of adipocytes, but also by a proliferation process which increases the number of adipocytes (10).

There are two different types of adipocytes within white adipose tissue: normal ‘white’ fat cells and ‘brite’ brown-in-white adipocytes. The latter express decoupled protein 1, a classic marker of brown fat cells, although they do not have the same molecular properties as brown fat cells (11). The distribution of these ‘brite’ adipocytes within white fat depots differed in mice (12). The different distribution of these ‘brite’ adipocytes in fat depots affects their metabolic processes, which could influence lipid release in adipose tissue (12). However, the authors do not mention any link with metabolic syndrome, and further investigations are necessary to evaluate a relationship between these ‘brite’ adipocytes and metabolic syndrome. To date, there is a lack of studies in horses and humans concerning this issue.

In addition to mature adipocytes, fat tissue contains a so-called stromal vascular fraction, which is located in loose connective tissue between fat cells and includes macrophages, fibroblasts, pericytes, mast cells, microvascular endothelial cells, and progenitor cells of the adipogenic line. Fat cells or mature adipocytes are of mesenchymal origin. The adipose tissue also constitutes a reservoir of mesenchymal stem cells, which may serve as an alternative cell source to bone marrow for tissue engineering in humans and equids (13, 14). White fat adipocytes are characterized by a single large fat droplet, which forces the nucleus to be squeezed into a thin rim at the periphery and a narrow hemline on the peripheral cytoplasm. The polygonal cells have a diameter of up to 120 μm and are embedded in a network of reticular fibers (collagen fibers of type III). White adipose tissue in humans is macroscopically divided by connective tissue septa into individual fat lobules, which are morphologically, functionally, and angiologically independent units. The areas of terminal circulation include an artery, which is usually situated in the axis of the lobule, while a paired vein collects blood on the surface. In contrast to former reports, the fat tissue is a highly perfused tissue (15, 16). Based on the cytoplasm area of fat cells, the capillary bed reaches a high level in a similar density to skeletal muscle. High vascularization of adipose tissue is important for the exchange of metabolites, high metabolic activity of individual fat cells, and its vital endocrine function (16). To the best of our knowledge, there is a lack of literature regarding whether and to what extent the horse differs in histology compared with humans.

The amount of fat tissue in the bodyweight of a normal adult human male is ∼8–20 and 21–33% in an adult female (17). Human fat is oily in consistency, semiliquid, and deep yellow at body temperature. The consistency of the fat depends on the melting point of fatty acids found in the fat tissue. A large proportion of fatty acid in adipose tissue in humans is oleic acid, a monounsaturated fatty acid that is assigned to omega 9 fatty acids based on the location of their double bond.

The amount of fat tissue in the bodyweight of a normal-weight horse is 5% (18). There are currently no studies focusing on differences in the amount of fat tissue in bodyweight according to breed, gender, or age. The white adipose tissue of the horse is also yellow and has an oily consistency. The yellow color is due to an increased incorporation of exogenous fat-soluble pigments, such as carotenoids (8). The most abundant fatty acid in the adipose tissue of the horse is also oleic acid (19).

In conclusion, the functional and structural condition of fat cells in horses and humans is widely comparable; therefore, a transfer of scientific knowledge from humans to equids might be possible. However, the percentage amount of body fat differs, which could lead to different amounts of metabolic products influencing the body system.

**Figure 1:**
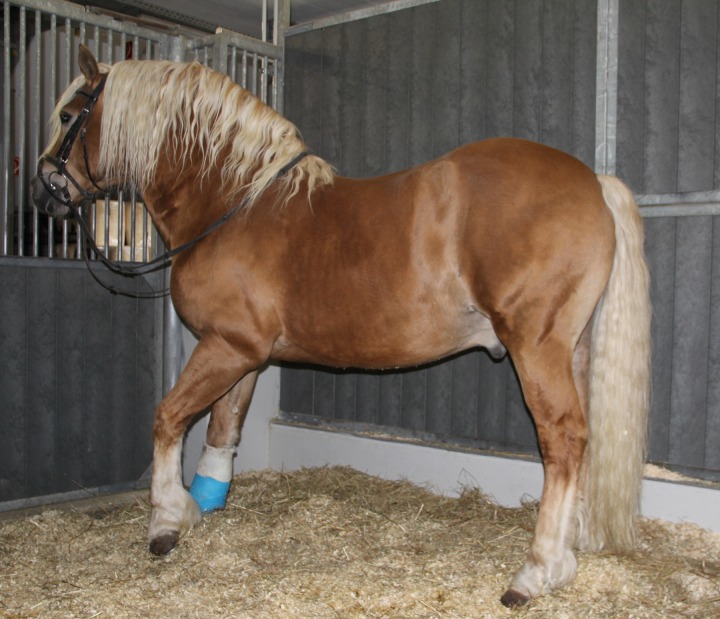
Horse suffering from EMS and laminitis. The horse shows distribution of fat at characteristic sites. The rear limbs are positioned forward to relieve stress from the front hooves, a condition associated with laminitis.

Horses with EMS present with a characteristic fat distribution showing deposits such as a cresty neck and at the comb, side of the chest, hip region, and tail head. Large omental fat deposits are also present, but not visible (Fig. 1).

Abdominal obesity is very common in humans, particularly males, suffering from HMS. Predominant fat accumulation in the femoral–gluteal region, commonly seen in females, is associated with a lower risk of metabolic disease (20, 21, 22).

The intra-abdominal fat accumulation in humans amounts to 10% of bodyweight and includes omental, mesenteric, and perirenal fat. Visceral fat surrounds internal organs (viscera) and is often used as a synonym for intra-abdominal fat (23). According to the definition of HMS corresponding to the International Diabetes Federation (IDF), the waist circumference has to be measured at ≥80 cm in women and ≥94 cm in men (http://www.idf.org, 09/08/2011). Mesenteric and omental fat is of greater importance in the development of insulin resistance, because their fatty acids and adipokines pass through the enterohepatic circulation. Additionally, omental fat has a more pronounced proinflammatory state than fat in other locations (24, 25). However, there are other important issues besides fat distribution which might influence the development of HMS. Humans with a reduced energy storage capacity in peripheral subcutaneous adipose tissue, for example, show a higher fat content in the liver, and skeletal and heart muscles. Consequently, they are predisposed to insulin resistance and developing type 2 diabetes mellitus (T2DM), an aspect that has to be investigated in horses (17).

Furthermore, a higher basal lipolysis has been detected in human adipocytes of omental and mesenteric adipose tissues, compared with adipocytes of subcutaneous fat tissue (26, 27, 28). The catecholamines stimulate lipolysis of omental fat tissue to a higher degree than in subcutaneous tissue, while insulin has a stronger inhibitory effect on subcutaneous adipose tissue (24, 27). These differences are partly due to the predominance of stimulating β-receptors over antilipolytic α-adrenoreceptors, and lower insulin receptor affinity of mesenteric omental adipocytes respectively (27, 29). In addition to differences in lipolysis at different localizations of adipose tissue, lipogenesis differs at various fat depots (30, 31, 32). There are also differences in gene expression in visceral and subcutaneous fat in humans; however, isolated and cultured human preadipocytes are still capable of their full and unique pattern of gene expression, regardless of their environment (33, 34, 35, 36). Further research is required, particularly in equines, to evaluate the importance of the site of fat deposition and differences in metabolism of these fat depots to assess their role in the development of metabolic syndrome.

Human adipose tissue produces adipokines, such as leptin, resistin, adiponectin, and visfatin. Furthermore, it releases inflammatory mediators, such as monocyte chemotactic protein 1 (MCP1 (CCL2)) and plasminogen activator inhibitor (PAI1 (SERPINE1)), as well as proinflammatory cytokines, such as tumor necrosis factor alpha (TNFα (TNF)), interleukin 1 (IL1 (IL1A)), IL6, and IL8 (CXCL8) (37, 38). Differences in the production of inflammatory mediators and their amount of expression in various fat tissues have also been reported. Adipose tissue around the nuchal ligament in horses is of particular importance in the production of inflammatory mediators, specifically IL1β and IL6. Furthermore, omental fat and fat in muscles are suspected of playing an important role in the development of a proinflammatory state, because expression of TNF is increased and suppression of cytokine signaling 3 (SOCS3) and Toll-like receptor 4 (TLR4) is significantly higher in comparison to subcutaneous fat in the area of the nuchal ligament (39, 40).

The gene expression of *IL1*, *IL6*, and *TNFα* was lower or not different in obese, hyperinsulinemic horses compared with euinsulinemic horses of normal weight (41). Moreover, no significant differences in gene expression of *TNFα*, *IL1β* (*IL1B*), *IL6*, *PAI1*, or *MCP1* mRNA were recognized in different fat depots in horses affected by EMS in comparison to healthy individuals (39). This is surprising, as an increase in ILs was assumed to be a causative factor in the pathogenesis of disease in previous reports (9, 42). However, a positive correlation of TNF expression, *IL1* mRNA, and body condition score (BCS) of horses was demonstrated in another study, in which an increased cytokine expression seemed to be a risk factor for the development of insulin resistance (9).

The importance of various inflammatory mediators and their patterns of expression in the horse have been controversially discussed. Marked differences seem to exist between humans and equines. Further studies are required to evaluate the reasons for these differences, as they may be a key factor in the pathogenesis of equine disease.

## Clinical signs

Metabolic syndrome affects all organ systems involved in metabolism to various degrees, and therefore, the disease results in an impaired energy metabolism of the entire organism (43).

Metabolic syndrome in humans is characterized by abdominal obesity, hypertension, dyslipidemia (hypertriglyceridemia, decreased HDL, and cholesterolemia), insulin resistance, increased oxidative burst, vascular dysfunction, increased coagulability (increased fibrinogen and tissue PAI1), and inflammation of adipose tissue (44, 45, 46, 47, 48, 49, 50, 51). All these factors contribute to a generalized proinflammatory state of the organism.

Symptoms of metabolic syndrome are used to identify humans with an increased risk of cardiovascular disease (52). Owing to endothelial dysfunction, affected individuals have an increased risk of developing atherosclerosis, coronary flow reserve is impaired, and there are independent associations among impaired coronary flow reserve, increased stiffness of the aorta, systolic blood pressure, and waist circumference (53, 54, 55). Symptoms of atherosclerosis are found in the entire body, including small vessels in the brain, extracranial carotid arteries, and coronary vessels. Possible consequences of atherosclerosis are ischemia, thrombosis, angina pectoris, heart attack, stroke, or sudden cardiac death (56).

Clinical signs in the horse include general (BCS ≥7/9; 57) or regional adiposity (cresty neck score ≥3/5), insulin resistance, a predisposition to laminitis, and enhanced oxidative burst (58, 59).

**Figure 2:**
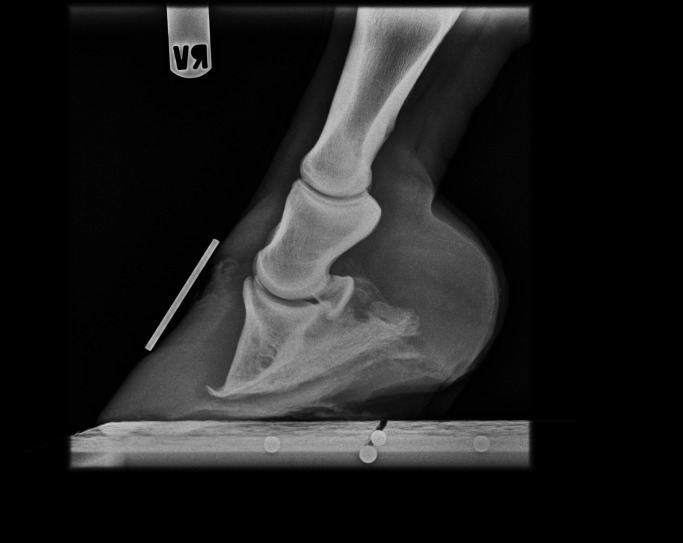
Lateromedial radiographic image of the right front limb of a horse showing rotation and evidence of sinking of the coffin bone.

**Figure 3:**
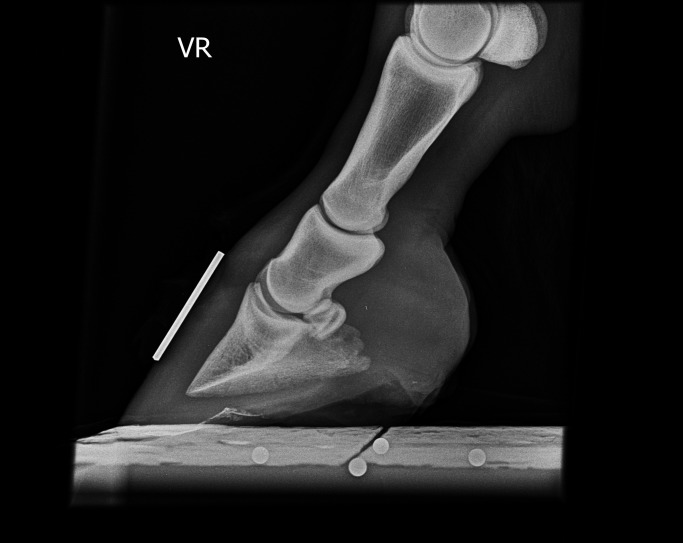
Lateromedial radiographic image of a physiological positioned hoof with no evidence of rotation or sinking of the coffin bone.

Laminitis, one of the most dangerous conditions in horses, ponies, and donkeys, might be the counterpart to central vascular dysfunction observed in humans. Severe or recurrent laminitis may limit the performance of the horse and may even result in euthanasia. Inflammation and ischemia of the digital dermal tissue lead to destruction of the interlaminar bond, which is the only support of the distal phalanx within the hoof capsule, and lameness, pedal bone rotation, and founder line formation follows (Figs 2 and 3) (7, 60). Further signs include hypertension, dyslipidemia, vascular dysfunction, and increased coagulation (58, 61, 62, 63, 64, 65).

Another consequence of HMS is non-alcoholic fatty liver disease. Pathological changes range from simple fatty infiltration to advanced non-alcoholic steatohepatitis and liver fibrosis (66, 67, 68). Studies on horses have not been carried out thus far, but elevated liver enzyme levels are occasionally found in affected horses, in particular γ-GT, corresponding to hepatic lipidosis detected in biopsy and necropsy specimens (7, 64).

A carcinogenic effect is also associated with metabolic syndrome in humans. It is assumed that females suffering from HMS have an increased risk of developing breast cancer (69). A predisposition to colon and rectal carcinoma is also discussed (70). In addition, a twofold risk of developing Barrett esophagus, a metaplastic transformation of the esophagus epithelium, has been reported (51). Horses suffering from EMS are suspected of developing intestinal lipoma at a younger age, but further investigation is warranted to confirm this hypothesis (71).

HMS is also associated with a higher prevalence of rheumatoid arthritis compared with the general population (72). Data in equines are missing.

Obese pregnant women have an increased risk of a variety of gestational and perinatal problems, such as gestational diabetes, fetal macrosomia, prematurity, birth defects, pre-eclampsia, eclampsia, increased caesarean section rate, and stillbirth (73, 74). A prolonged estrous cycle is observed in obese mares (9).

T2DM is of particular concern in humans suffering from metabolic syndrome, and it may also develop in horses in pronounced cases of EMS. At the time of diabetes diagnosis, insulin resistance and relative insulin deficiency may be present. Type 2 is the most common type of DM in humans, accounting for about 90% of cases (http://www.dft.org//types-diabetes). T2DM in horses may be more common than generally considered and is the main diabetes type observed in horses as well. Moreover, T2DM is the end stage of EMS (71, 75).

Several risk factors have been associated with T2DM and include a family history of diabetes, overweight, dietary factors, physical inactivity, increasing age, high blood pressure, ethnicity, impaired glucose tolerance, history of gestational diabetes, and poor nutrition during pregnancy (http://www.dft.org//types-diabetes).

Various international research centers have been involved in identifying genes predisposed to T2DM in humans. An association of T2DM with the calpain 10 (*CAPN10*) gene was initially identified, and later, its association with the transcription factor 7-like 2 (*TCF7L2*) gene, whose genetic variants in affected individuals increase the risk of diabetes by ∼1.5 times (76, 77).

The most common risk factors in horses include metabolic syndrome and PPID. Unfortunately, studies on equine medicine are lacking concerning genetic issue or other predisposing factors compared with human medicine.

In summary, a significant clinical overlap exists between both species. The most interesting question seems to be whether a comparable genesis exists for cardiovascular disease in humans and laminitis in equines.

## Pathophysiology of metabolic syndrome

Multiple hypotheses regarding the pathogenesis of metabolic syndrome exist in human and equine medicine. Some of them can be supported by evidence, while others require further research.

One of these hypotheses regarding the development of metabolic syndrome is the regulation of bodyweight through the ponderostat. The ponderostat is a control or supervisory point within the brain, probably located in the hypothalamus, responsible for the regulation of bodyweight. Based on the regulation theory of cybernetics (78), the ponderostat constantly compares the actual value of the organism with the set point and responds to weight loss with physical signals, such as hunger. The mechanisms of the ponderostat are still poorly understood, but probably do not depend directly on energy supply. The regulation represents a very complex process, which is influenced partly by the immune system (79). The hormones leptin and oleoyl-estrone were postulated as ponderostat signals. As the secretion capacity of leptin differs within different fat depots depending on the age, gender, and circadian rhythm, the likelihood that leptin represents a true ponderostat signal is low (79, 80, 81, 82). Recent studies have demonstrated that oleoyl-estrone itself cannot be responsible for the control of body fat, but a derivate might (83). In other words, the brain is involved in the pathogenesis of metabolic syndrome, but the exact mechanisms remain obscure.

White adipose tissue plays a key role in the development of metabolic syndrome. Owing to an excessive supply of energy, adipocytes increase in size causing ‘stress’ in adipose tissue. During expansion of hypertrophic adipocytes, a signaling cascade is initiated, leading to the remodeling of tissue and recruitment of inflammatory cells. Owing to proinflammatory signals in adipose tissue, there is an increased activity of macrophages, which leads to further inflammation of adipose tissue. Persistent inflammation leads to accelerated lipolysis and an increased amount of free fatty acids in the bloodstream (84). Inflammation of adipose tissue induces production of proinflammatory adipokines that increase their own synthesis and synthesis of other systemic inflammatory markers and *vice versa*. Consequently, secondary synthesis of acute-phase proteins in the liver is induced (37). The result is the development of an inflammatory condition that becomes chronic, as causative excessive obesity cannot be eliminated by the immune system (43). This chronic disease does not follow a classic pattern and leads to a variety of pathological events that have still not been investigated in detail.

One of the major pathological events of metabolic syndrome in both species is insulin resistance. On the one hand, this is caused by inhibition of insulin signal transmission pathways by adipokines and cytokines and, on the other hand, by accumulation of intracellular fat in insulin-sensitive tissue, such as skeletal muscle, liver, and pancreas (85, 86).

Oxidative stress is also involved in the development of insulin resistance. Highly reactive toxic oxygen and nitric oxide (NO) radicals, which occur frequently in mitochondrial weakness, induce cytocidal changes to receptor proteins influencing their functions (87). Holbrook *et al*. (41) found significant increases in neutrophil oxidative stress in obese horses with hyperinsulinemia. One receptor protein that might be damaged through oxidative burst is glucose transporter 4 (GLUT4 (SLC2A4)). This receptor is one of the insulin-dependent GLUTs responsible for the uptake of glucose into cells and is significantly less expressed on the cell surface of muscle and adipose tissue of insulin-resistant horses (88).

The insulin receptor substrate 1 (IRS1), a substrate of insulin receptor tyrosine kinase, gained increased interest in human medicine as it plays a central role in the insulin-stimulated signal transduction pathway (89, 90, 91, 92, 93, 94). However, Waller *et al*. (40) found no difference in total content or serine phosphorylated IRS1 sampled from visceral and subcutaneous adipose tissue and skeletal muscle biopsies in horses with insulin resistance compared with healthy subjects.

It is known that insulin resistance in muscle and adipose tissue in humans leads to flooding of the liver with free fatty acids, causing an increased triglyceride and very-LDL synthesis in hepatocytes (95). The result is fatty degeneration of the liver, increased synthesis of C-reactive protein, fibrinogen, coagulation factors, and angiotensinogen. The liver plays a major role in HMS regarding the development of coagulation disorders, thrombosis, vascular occlusion, and inflammation (96, 97).

Not only do free fatty acids lead to fatty liver degeneration, but there is also a correlation with disturbance in the intestinal microflora in HMS. Owing to bacterial overgrowth and increased permeability of the intestine, a greater amount of endotoxins and bacterial DNA is transported to the liver via the portal vein. Once they reach the liver, inflammation is induced due to activation of the TLR (especially TLR4 and TLR9), a receptor of the innate immune system, which potentiates expression and secretion of proinflammatory cytokines. This leads to the development of fatty liver degeneration and, in turn, as well as in addition, this may induce insulin resistance in insulin-sensitive tissues (98, 99, 100).

It is assumed that there is a correlation between TLR4 and downregulation of insulin response via SOCS3. Cytokine signaling 3 is able to inhibit insulin signaling pathways through leptin signaling in the hypothalamus, as well as to inhibit insulin signaling pathways in the adipose tissue and liver, which lead indirectly to insulin resistance of peripheral organs (40).

Additionally, an increased expression of the SOCS may be caused by proinflammatory cytokines (101, 102). An increase in SOCS3 protein in rodents leads to impaired insulin response in the liver, adipose tissue, and skeletal muscle (89, 103, 104). Whether this occurs in horses and humans also requires further research.

A vicious circle exists in humans due to magnesium deficiency, which may create an increasing excretion of magnesium through the kidney induced by hyperglycemia and hyperinsulinemia. This leads through different pathways to hypertension, glucose intolerance, and hyperlipidemia, which additionally increases clearance of the electrolyte in the kidney (105, 106). It remains to be elucidated whether a magnesium deficiency in EMS exists and if there is an association with laminitis. Magnesium deficiency may lead to changes in the intracellular Ca^2+^/Mg^2+^ ratio, which induces an increase in vascular tone and increased secretion of catecholamines through sympathetic nerve endings, and this may ultimately lead to hypoperfusion in the vascular bed of the hoof (105, 106, 107).

The most important and life-threatening feature in humans is atherosclerosis, which is characterized by chronic progressive degeneration of arteries with vascular wall remodeling by connective tissue proliferation, intra- and extracellular deposits of cholesterol, fatty acids, and lime, as well as accumulation of collagen and proteoglycans. All these lead to increased vascular stiffness and constriction. Two major hypotheses have been discussed in the literature in recent decades to explain this process: ‘response to injury’ and ‘lipoprotein-induced atherosclerosis’ (108, 109, 110).

The ‘response to injury’ hypothesis is based on the mechanical damage of the endothelial cell layer due to trauma, high blood pressure, biochemical damage caused by bacterial toxins, viral infections, or immune complex formation and biophysical injury at the molecular level. Consequently, growth factors and cytokines stimulate proliferation and migration of smooth muscle cells from multilayer media into intima; fat deposition leads to formation of foam cells, fat-laden immune cells of macrophage type, in the intima and media, which leads to plaque formation. A modification of this hypothesis is based on the suspected endothelial dysfunction caused by a singular injury or by a gradually occurring imbalance of endothelial function. The ‘lipoprotein-induced atherosclerosis’ hypothesis is based on a rapid uptake of chemically modified LDL by macrophages and subsequent conversion into foam cells. This hypothesis involves injury of endothelial cells only as a partial step in a sequence of complex operations (108, 109, 110).

Based on current knowledge, atherosclerosis does not occur in horses, but it remains to be evaluated why arteriosclerosis is not a feature of EMS. It has to be considered that there is a different amount of HDL and LDL cholesteryl esters in horses compared with humans. There is no plasma cholesteryl ester transfer protein in horses and there is only a small percentage of LDL cholesteryl esters derived from HDL cholesteryl esters. However, LDLs are responsible for considering the ‘lipoprotein-induced atherosclerosis’ hypothesis (111). Horses, on average, do not reach the age of humans. However, there are horses, especially ponies, which reach an age of up to 50 years without any presentation of atherosclerosis. Another difference is diet: horses are usually strict vegetarians. Further research is required to answer specific questions, such as: what prevents the horse from having this aspect of metabolic syndrome, and could this mechanism be used as a preventative measure in humans?

Little is known about the pathogenesis of laminitis and its association with EMS (112). Damage to the lamellar (dermo-epidermal) interface in the horse's hoof can result in structural changes, such as distal phalanx disorientation and lameness, both of which are defining features of laminitis (113, 114). However, the role of inflammation, as well as the mechanism leading to hypoperfusion and damage to the endothelium of vessels of lamellar tissue in hyperinsulinemic laminitis, has not been adequately defined. The basic principles of endothelial damage may be partly identical to humans. However, the consequence of this endothelial damage in the vessels of the hoof seems to be liable to other mechanisms, because it does not result in the same structural changes observed in arteriosclerosis (42, 60, 113, 114, 115).

It has already been mentioned that the mechanism leading to injury of the endothelium of vessels, or rather clinical onset of laminitis, is the consequence of a chronic mild generalized inflammatory reaction with a concomitant increase in oxidative stress. This is characterized by an increased activity of the NAD(P)H oxidase, which leads to increased production of reactive oxygen radicals. Furthermore, there is a decreased activity of superoxide dismutase, glutathione peroxidase, and heme oxygenase 2, which represent the key enzymes of the antioxidant defense system. Their impairment can result in damage and endothelial dysfunction of blood vessels in the hoof (50).

Another hypothesis is based on the increased coagulatory state in blood vessels of the hoof, due to increased expression of coagulation factors in the liver, which can ultimately lead to hypoperfusion through to ischemia (96, 97).

Induction of hyperglycemia followed by endogenous hyperinsulinemia in horses was associated with histopathological evidence of laminitis (116).

Histopathological changes early in disease progression included a decreased secondary epidermal lamellar (SEL) width and increased histomorphological evidence of SEL basal (and suprabasal) cell death. Increased cellular proliferation in SELs, infiltration of the dermis with small numbers of leucocytes, and basement membrane (BM) damage occurred later. Some lesions, such as narrowing of the SELs, were progressive over time. Cellular pathology preceded leucocyte infiltration and BM pathology, indicating that the latter changes may be secondary or downstream events in hyperinsulinemic laminitis (116).

Assumptions have been made that hyperinsulinemia in EMS is due to the vasoregulatory properties of insulin. Insulin is able to reduce the synthesis of nitric oxide, which is an important vasodilator. However, there is no effect of insulin on the synthesis of MAPK, which induces vasoconstriction. In combination, the result is a superior drift to vasoconstriction, which could play an important role in the pathogenesis of laminitis (114, 115, 116, 117, 118, 119, 120, 121).

Another potential mechanism by which insulin-induced laminitis develops may be significant in endothelin receptor (ETR) expression (121, 122). Endothelin 1 (ET1) is a potent vasoconstrictor produced by vascular endothelial cells binding to at least two receptors, ETRA (EDNRA) and ETRB (EDNRB) (123). ETRB is located primarily in the endothelium, and activation of ETRB removes ET1 from the circulation, thereby resulting in vasodilation (124, 125). ETRA is located primarily in vascular smooth muscle, and activation of ETRA in human patients causes cell growth and contraction of smooth muscle cells, resulting in hypertension (125). A study by Gauff *et al*. (122) indicated that localization and expression of ETRA and ETRB varied within lamellar tissue of the equine forelimb. The results of the study suggested that the vasoconstrictive effect of hyperinsulinemia is caused primarily by activation of ETRA located in smooth muscle of blood vessels (122).

In addition, insulin-like growth factor 1 (IGF1), a polypeptide with close structural homology to insulin and well-known effects in terms of activating cell proliferation and tissue growth and repair, might be another key in the pathogenesis of laminitis (126).

Gene expression for IGF1 receptor (IGF1R) and insulin receptor were decreased by 13- to 32-fold during a prolonged euglycemic, hyperinsulinemic clamp test during mid-developmental and acute phases of insulin-induced laminitis. There was no increase in serum IGF1 concentrations during prolonged euglycemic, hyperinsulinemic clamp, consistent with downregulation of both receptors by insulin. Stimulation of IGF1R by insulin may lead to inappropriate lamellar epidermal cell proliferation and lamellar weakening, a potential mechanism for hyperinsulinemic laminitis. Targeting this receptor may provide insights into the pathogenesis or identify a novel therapy for hyperinsulinemic laminitis (127, 128).

The development of laminitis may also follow an increased activity of TLR4, as part of the generalized chronic low-grade inflammatory reaction, which is associated with downregulation of insulin response via SOCS3 and is responsible for increased expression and secretion of proinflammatory cytokines. Both pathways lead indirectly to insulin resistance and damage of endothelial cells. TLR4 is gaining increasing attention and its activity may serve as an early marker of insulin resistance in the horse (71, 129). Another indicator of chronic inflammation in the vascular bed of the hoof is increased expression of TNFα, which also promotes insulin resistance. An increase in this inflammatory marker could be assessed in ponies with hyperinsulinemia and previous laminitis (58).

As is commonly known, metalloproteinases play an important role in weakening lamellar tissue during laminitis. Therefore, it is of interest if there is an increased activity of these metalloproteinases in lamellar tissue in horses suffering from hyperinsulinemia.

It seems most likely that the pathogenesis of laminitis in EMS is based on multiple factors. At present, no definite evidence exists as to what extent proinflammatory conditions, coagulative changes, and disruption of insulin pathways are involved in the development of the disease.

## Summary

Metabolic syndrome in humans differs in several aspects from the equine disease. The most important pathological factor in humans is affection of the cardiovascular system, and in horses, the development of laminitis. The mechanisms that lead to these potentially life-limiting consequences are not fully comparable, although the changes in both species take place in the vascular system. However, inflammatory conditions in adipose tissue and effects on metabolic and biochemical processes show similarities between both species.

## Funding 

This review did not receive any specific grant from any funding agency in the public, commercial or not-for-profit sector.

## References

[1] Camus JP (1966). Gout, diabetes, hyperlipemia: a metabolic trissyndrome. Revue du Rhumatisme et des Maladies Ostéo Articulaires.

[2] Reaven GM (1988). Banting Lecture 1988: Role of insulin resistance in human disease. Diabetes.

[3] Kaplan NM (1989). The deadly quartet. Upper-body obesity, glucose intolerance, hypertriglyceridemia, and hypertension. Archives of Internal Medicine.

[4] National Cholesterol Education Program (NCEP) Expert Panel on Detection, Evaluation, and Treatment of High Blood Cholesterol in Adults (Adult Treatment Panel III) (2002). Third Report of the National Cholesterol Education Program (NCEP) Expert Panel on Detection, Evaluation, and Treatment of High Blood Cholesterol in Adults (Adult Treatment Panel III) final report. Circulation.

[5] Johnson PJ (2002). The equine metabolic syndrome peripheral Cushing's syndrome. Veterinary Clinics of North America. Equine Practice.

[6] Fulop T, Tessier D, Carpentier A (2006). The metabolic syndrome. Pathologie–Biologie.

[7] Frank N, Goer RJ, Bailey SR, Durham AE, Johnson PJ (2010). Equine metabolic syndrome. Journal of Veterinary Internal Medicine.

[8] Liebich HG. Funktionelle Histologie der Haussugetiere. In *Lehrbuch und Farbatlas fr Praxis und Studium* [Functional histology of house mammals: textbook and color atlas of practice and study]. 5th Edn, pp 77–79. Schattauer GmbH: Stuttgard, Germany, 2010.

[9] Vick MM, Sessions DR, Murphy BA (2006). Obesity is associated with altered metabolic and reproductive activity in the mare: effects of metformin on insulin sensitivity and reproductive cyclicity. Reproduction, Fertility, and Development.

[10] Schwegler JS & Lucius R. Bindeund Stützgewebe. In *Der Mensch Anatomie und Physiologie* [Human Anatomy and Physiology], 5th edn. Thieme Verlag: Stuttgart, Germany, 2011.

[11] Petrovic N, Walden TB, Shabalina IG, Timmons JA, Cannon B, Nedergaard J (2010). Chronic peroxisome proliferator-activated receptor γ (PPARγ) activation of epididymally derived white adipocyte cultures reveals a population of thermogenically competent, UCP1-containing adipocytes molecularly distinct from classic brown adipocytes. Journal of Biological Chemistry.

[12] Walden TB, Hansen IR, Timmons JA, Cannon B, Nedergaard J (2012). Recruited versus nonrecruited molecular signatures of brown, ‘brite’ and white adipose tissues. American Journal of Physiology. Endocrinology and Metabolism.

[13] Zuk PA, Zhu M, Mizuno H, Huang J, Futrell JW, Katz AJ, Benhaim P, Lorenz HP, Hedrick MH (2001). Multilineage cells from human adipose tissue: implications for cell-based therapies. Tissue Engineering.

[14] Koch TG, Berg LC, Betts DH (2008). Concepts for the clinical use of stem cells in equine medicine. Canadian Veterinary Journal.

[15] Crandall DL, Hausman GJ, Kral JG (1997). A review of the microcirculation of adipose tissue: anatomic, metabolic, and angiogenic perspectives. Microcirculation.

[16] Ballard KW (1978). Functional characteristics of the microcirculation in white adipose tissue. Microvascular Research.

[17] Gallagher D, Kelley DE, Yim JE, Spence N, Albu J, Boxt L, Pi-Sunyer FX, Heshka S (2009). Adipose tissue distribution is different in type 2 diabetes. American Journal of Clinical Nutrition.

[18] Litwinczuk A, Florek M, Skalecki P, Litwinczuk Z (2007). Slaughter value and quality of horse carcasses in relation to the pre-slaughter body weight. Fleischwirtschaft International.

[19] Souci S, Fachmann W, Kraut H. Food composition and nutrition tables. In *Deutsche Forschungsanstalt für Lebensmittelchemie*. 7th Edn. Wissenschaftliche Verlagsgesellschaft: Stuttgart, Germany, 2008.

[20] Misra A, Garg A, Abate N, Peshock RM, Stray-Gundersen J, Grundy SM (1997). Relationship of anterior and posterior subcutaneous abdominal fat to insulin sensitivity in nondiabetic men. Obesity Research.

[21] Snijder MB, Dekker JM, Visser M, Bouter LM, Stehouwer CD, Kostense PJ, Yudkin JS, Heine RJ, Nijpels G, Seidell JC (2003). Associations of hip and thigh circumferences independent of waist circumference with the incidence of type 2 diabetes: the Hoorn study. American Journal of Clinical Nutrition.

[22] Tanko LB, Bagger YZ, Alexandersen P, Larsen PJ, Christiansen C (2003). Peripheral adiposity exhibits an independent dominant antiatherogenic effect in elderly women. Circulation.

[23] Item F, Konrad D (2012). Visceral fat and metabolic inflammation: the portal theory revisited. Obesity Reviews.

[24] Santosa S, Jensen MD (2008). Why are we shaped differently, and why does it matter?. American Journal of Physiology. Endocrinology and Metabolism.

[25] O'Rourke RW, Metcalf MD, White AE, Madala A, Winters BR (2009). Depot-specific differences in inflammatory mediators and a role for NK cells and IFN-γ in inflammation in human adipose tissue. International Journal of Obesity.

[26] Ostman J, Arner P, Engfeldt P, Kager L (1979). Regional differences in the control of lipolysis in human adipose tissue. Metabolism.

[27] Björntorp P (1990). ‘Portal’ adipose tissue as a generator of risk factors for cardiovascular disease and diabetes. Arteriosclerosis.

[28] Arner P (1998). Not all fat is alike. Lancet.

[29] Bolinder J, Kager L, Ostman J, Arner P (1983). Differences at the receptor and postreceptor levels between human omental and subcutaneous adipose tissue in the action of insulin on lipolysis. Diabetes.

[30] Edens NK, Fried SK, Kral JG, Hirsch J, Leibel RL (1993). *In vitro* lipid synthesis in human adipose tissue from three abdominal sites. American Journal of Physiology.

[31] Caesar R, Manieri M, Kelder T, Boekschoten M, Evelo C, Müller M, Kooistra T, Cinti S, Kleemann R, Drevon CA (2010). A combined transcriptomics and lipidomics analysis of subcutaneous, epididymal and mesenteric adipose tissue reveals marked functional differences. PLoS ONE.

[32] Koutsari C, Ali AH, Mundi MS, Jensen MD (2011). Storage of circulating free fatty acid in adipose tissue of postabsorptive humans: quantitative measures and implications for body fat distribution. Diabetes.

[33] Vohl MC, Sladek R, Robitaille J, Gurd S, Marceau P, Richard D, Hudson TJ, Tchernof A (2004). A survey of genes differentially expressed in subcutaneous and visceral adipose tissue in men. Obesity Research.

[34] Gesta S, Tseng YH, Kahn CR (2007). Developmental origin of fat: tracking obesity to its source. Cell.

[35] Tchkonia T, Lenburg M, Thomou T, Giorgadze N, Frampton G, Pirtskhalava T, Cartwright A, Cartwright M, Flanagan J, Karagiannides I (2007). Identification of depot-specific human fat cell progenitors through distinct expression profiles and developmental gene patterns. American Journal of Physiology. Endocrinology and Metabolism.

[36] Yamamoto Y, Gesta S, Lee KY, Tran TT, Saadatirad P, Kahn CR (2010). Adipose depots possess unique developmental gene signatures. Obesity.

[37] Rasouli N, Kern PA (2008). Adipocytokines and the metabolic complications of obesity. Journal of Clinical Endocrinology and Metabolism.

[38] Ouchi N, Parker JL, Lugus JJ, Walsh K (2011). Adipokines in inflammation and metabolic disease. Nature Reviews. Immunology.

[39] Burns TA, Geor RJ, Mudge LJ, Mc Cutcheon KW, Hinchcliff KW, Belknap JK (2010). Proinflammatory cytokine and chemokine gene expression profiles in subcutaneous and visceral adipose tissue depots of insulin-resistant and insulin-sensitive light breed horses. Journal of Veterinary Internal Medicine.

[40] Waller AP, Huettner L, Kohler K, Lacombe VA (2012). Novel link between inflammation and impaired glucose transport during equine insulin resistance. Veterinary Immunology and Immunopathology.

[41] Holbrook TC, Tipton T, McFarlane D (2012). Neutrophil and cytokine dysregulation in hyperinsulinemic obese horses. Veterinary Immunology and Immunopathology.

[42] Treiber K, Carter R, Gay L, Williams C, Geor R (2009). Inflammatory and redox status of ponies with a history of pasture-associated laminitis. Veterinary Immunology and Immunopathology.

[43] Alemany M (2013). Relationship between energy dense diets and white adipose tissue inflammation in metabolic syndrome. Nutrition Research.

[44] Landin K, Tengborn L, Smith U (1990). Elevated fibrinogen and plasminogen activator inhibitor (PAI-1) in hypertension are related to metabolic risk factors for cardiovascular disease. Journal of Internal Medicine.

[45] Bjorntorp P (1991). Metabolic implications of body fat distribution. Diabetes Care.

[46] Watarai T, Yamasaki Y, Ikeda M, Kubota M, Kodama M, Tsujino T (1999). Insulin resistance contributes to carotid arterial wall thickness in patients with non-insulin-dependent diabetes mellitus. Endocrine Journal.

[47] Hamaguchi M, Kojima T, Takeda N, Nakagawa T, Taniguchi H, Fujii K, Omatsu T, Nakajima T, Sarui H, Shimazaki M (2005). The metabolic syndrome as a predictor of nonalcoholic fatty liver disease. Annals of Internal Medicine.

[48] Aquilante CL, Kosmiski SD, Zineh I (2008). Relationship between plasma resistin concentrations, inflammatory chemokines, and components of the metabolic syndrome in adults. Metabolism.

[49] Hung J, McQuillan BM, Thompson PL, Beilby JP (2008). Circulating adiponectin levels associate with inflammatory markers, insulin resistance and metabolic syndrome independent of obesity. International Journal of Obesity.

[50] Roberts CK, Sindhu KK (2009). Oxidative stress and metabolic syndrome. Life Sciences.

[51] Leggett CL, Nelsen EM, Tian J, Schleck CB, Zinsmeister AR, Dunagan KT, Locke GR, Wang KK, Talley NJ, Iyer PG (2013). Metabolic syndrome as a risk factor for barrett esophagus: a population-based case–control study. Mayo Clinic Proceedings.

[52] Ma X, Zhu S (2013). Metabolic syndrome in the prevention of cardiovascular diseases and diabetes–still a matter of debate?. European Journal of Clinical Nutrition.

[53] Celermajer DS (1997). Endothelial dysfunction: does it matter? Is it reversible?. Journal of the American College of Nutrition.

[54] Bonetti PO, Lerman LO, Lerman A (2003). Endothelial dysfunction: a marker of atherosclerotic risk. Arteriosclerosis, Thrombosis, and Vascular Biology.

[55] Tok D, Ozcan F, Kadife I, Turak O, Cağlı K, Başar N, Gölbaşı Z, Aydoğdu S (2013). Impaired coronary flow reserve evaluated by echocardiography is associated with increased aortic stiffness in patients with metabolic syndrome: an observational study. Anadolu Kardiyoloji Dergisi.

[56] Ohnuki T, Takahashi W, Ohnuki Y, Kawada S, Takizawa S (2013). Significance of the presence of metabolic syndrome in patients with asymptomatic arteriosclerosis affecting the aorta and the cerebral, extra-cranial carotid and coronary arteries. Internal Medicine.

[57] Henneke DR, Potter GD, Kreider JL, Yeates BF (1983). Relationship between condition score, physical measurements and body fat percentage in mares. Equine Veterinary Journal.

[58] Carter RA, Treiber KH, Geor RJ, Douglass L, Harris PA (2009). Prediction of incipient pasture-associated laminitis from hyperinsulinaemia, hyperleptinaemia and generalised and localised obesity in a cohort of ponies. Equine Veterinary Journal.

[59] Carter RA, Geor RJ, Burton Staniar W, Cubitt TA, Harris PA (2009). Apparent adiposity assessed by standardised scoring systems and morphometric measurements in horses and ponies. Veterinary Journal.

[60] Katz LM, Bailey SR (2012). A review of recent advances and current hypotheses on the pathogenesis of acute laminitis. Equine Veterinary Journal.

[61] Gentry LR, Thompson DL, Gentry GT (2002). The relationship between body condition, leptin, and reproductive and hormonal characteristics of mares during the seasonal anovulatory period. Journal of Animal Science.

[62] Cartmill JA, Thompson DL, Storer WA (2003). Endocrine responses in mares and geldings with high body condition scores grouped by high vs. low resting leptin concentrations. Journal of Animal Science.

[63] Houseknecht KL, Spurlock ME (2003). Leptin regulation of lipid homeostasis: dietary and metabolic implications. Nutrition Research Reviews.

[64] Frank N, Elliott SB, Brandt LE (2006). Physical characteristics, blood hormone concentrations, and plasma lipid concentrations in obese horses with insulin resistance. Journal of the American Veterinary Medical Association.

[65] Lamounier-Zepter V, Ehrhart-Bornstein M, Bornstein SR (2006). Insulin resistance in hypertension and cardiovascular disease. Best Practice & Research. Clinical Endocrinology & Metabolism.

[66] Eckel RH, Grundy SM, Zimmet PZ (2005). The metabolic syndrome. Lancet.

[67] Alberti KG, Eckel RH, Grundy SM, Zimmet PZ, Cleeman JI, Donato KA, Fruchart JC, James WP, Loria CM, Smith SC (2009). Harmonizing the metabolic syndrome: a joint interim statement of the International Diabetes Federation Task Force on Epidemiology and Prevention; National Heart, Lung, and Blood Institute; American Heart Association; World Heart Federation; International Atherosclerosis Society; and International Association for the Study of Obesity. Circulation.

[68] Torres do Rego A, Perez de Isla L, Saltijeral Cerezo A, Vitale G, Izarra A, Alvarez-Sala Walther LA (2014). Cholesterol control according to the presence of metabolic syndrome in coronary and diabetic patients. Relationship with non-alcoholic fatty liver disease. European Journal of Internal Medicine.

[69] Alokail MS, Al-Daghri N, Abdulkareem A, Draz HM, Yakout SM, Alnaami AM, Sabico S, Alenad AM, Chrousos GP (2013). Metabolic syndrome biomarkers and early breast cancer in Saudi women: evidence for the presence of a systemic stress response and/or a pre-existing metabolic syndrome-related neoplasia risk?. BMC Cancer.

[70] Aleksandrova K, Nimptsch K, Pischon T (2013). Influence of obesity and related metabolic alterations on colorectal cancer risk. Current Nutrition Reports.

[71] Frank N, Reed SM, Bayly WM & Sellon DC. Insulin resistance and equine metabolic syndrome. In *Equine Internal Medicine*, 3rd edn, pp 1270–1277. Saunders/Elsevier: Philadelphia, PA, USA, 2010.

[72] Cojocaru M, Cojocaru IM, Silosi I, Vrabie CD (2012). Metabolic syndrome in rheumatoid arthritis. Maedica.

[73] Cedergren MI (2004). Maternal morbid obesity and the risk of adverse pregnancy outcome. Obstetrics and Gynecology.

[74] Leeners B, Rath W, Kuse S, Irawan C, Imthurn B, Neumaier-Wagner P (2006). BMI: new aspects of a classical risk factor for hypertensive disorders of pregnancy. Clinical Science.

[75] Durham AE, Hughes KJ, Cottle HJ, Rendle DI, Boston RC (2009). Type 2 diabetes mellitus with pancreatic β cell dysfunction in 3 horses confirmed with minimal model analysis. Equine Veterinary Journal.

[76] Grant SF, Thorleifsson G, Reynisdottir I, Benediktsson R, Manolescu A, Sainz J, Helgason A, Stefansson H, Emilsson V, Helgadottir A (2006). Variant of transcription factor 7-like 2 (TCF7L2) gene confers risk of type 2 diabetes. Nature Genetics.

[77] Ahlqvist E, Ahluwalia TS, Groop L (2011). Genetics of type 2 diabetes. Clinical Chemistry.

[78] Cabanac M (2001). Regulation and the ponderostat. International Journal of Obesity.

[79] Alemany M (2012). Steroid hormones interrelationships in the metabolic syndrome: an introduction to the ponderostat hypothesis. Hormones.

[80] Hube F, Lietz U, Igel M, Jensen PB, Tornqvist H, Joost HG, Hauner H (1996). Difference in leptin mRNA levels between omental and subcutaneous abdominal adipose tissue from obese humans. Hormone and Metabolic Research.

[81] Saad MF, Damani S, Gingerich RL, Riad-Gabriel MG, Khan A, Boyadjian R, Jinagouda SD, el-Tawil K, Rude RK, Kamdar V (1997). Sexual dimorphism in plasma leptin concentration. Journal of Clinical Endocrinology and Metabolism.

[82] Gómez Abellán P, Gómez Santos C, Madrid JA, Milagro FI, Campion J, Martínez JA, Luján JA, Ordovás JM, Garaulet M (2011). Site-specific circadian expression of leptin and its receptor in human adipose tissue. Nutrición Hospitalaria.

[83] Vilà R, Cabot C, Villarreal L, Monegal A, Ayet E, Romero MM, Grasa MM, Esteve M, Fernández-López JA, Remesar X (2011). Oleoyl-estrone is a precursor of an estrone-derived ponderostat signal. Journal of Steroid Biochemistry and Molecular Biology.

[84] Manteiga S, Choi K, Jayaraman A, Lee K (2013). Systems biology of adipose tissue metabolism: regulation of growth, signaling and inflammation. Wiley Interdisciplinary Reviews. Systems Biology and Medicine.

[85] Summers SA (2006). Ceramides in insulin resistance and lipotoxicity. Progress in Lipid Research.

[86] Kashyap SR, Defronzo RA (2007). The insulin resistance syndrome: physiological considerations. Diabetes & Vascular Disease Research.

[87] De la Monte S, Wands JR (2006). Molecular indices of oxidative stress and mitochondrial dysfunction occur early and often progress with severity of Alzheimer`s disease. Journal of Alzheimer's Disease.

[88] Waller AP, Burns TA, Mudge MC, Belknap JK, Lacombe VA (2011). Insulin resistance selectively alters cell-surface glucose transporters but not their total protein expression in equine skeletal muscle. Journal of Veterinary Internal Medicine.

[89] Yaspelkis BB, Kvasha IA, Figueroa TY (2009). High-fat feeding increases insulin receptor and IRS-1 coimmunoprecipitation with SOCS-3, IKKα/β phosphorylation and decreases PI-3 kinase activity in muscle. American Journal of Physiology. Regulatory, Integrative and Comparative Physiology.

[90] Hotamisligil GS, Murray DL, Choy N, Spiegelman BM (1994). Tumor necrosis factor α inhibits signaling from the insulin receptor. PNAS.

[91] Aguirre V, Werner ED, Giraud J, Lee YH, Shoelson SE, White MF (2002). Phosphorylation of Ser307 in insulin receptor substrate-1 blocks interactions with the insulin receptor and inhibits insulin action. Biological Chemistry.

[92] Li J, Yu X, Pan W, Unger RH (2002). Gene expression profile of rat adipose tissue at the onset of high-fat-diet obesity. American Journal of Physiology. Endocrinology and Metabolism.

[93] Hotamisligil GS (2003). Inflammatory pathways and insulin action. International Journal of Obesity and Related Metabolic Disorders.

[94] De Luca C, Olefsky JM (2008). Inflammation and insulin resistance. FEBS Letters.

[95] Alexander CM, Landsman PB, Teutsch SM, Haffner SM, Third National Health and Nutrition Examination Survey (NHANES III), National Cholesterol Education Program (NCEP) (2003). NCEP-defined metabolic syndrome, diabetes, and prevalence of coronary heart disease among NHANES III participants age 50 years and older. Diabetes.

[96] Devaraj S, Rosenson RS, Jialal I (2004). Metabolic syndrome: an appraisal of the proinflammatory and procoagulant status. Endocrinology and Metabolism Clinics of North America.

[97] Krauss RM, Siri PW (2004). Metabolic abnormalities: triglyceride and low-density lipoprotein. Endocrinology and Metabolism Clinics of North America.

[98] Tsukumo DM, Carvalho-Filho MA, Carvalheira JB, Prada PO, Hirabara SM, Schenka AA, Araújo EP, Vassallo J, Curi R, Velloso LA (2007). Loss-of function mutation in Toll-like receptor 4 prevents diet-induced obesity and insulin resistance. Diabetes.

[99] Saberi M, Woods NB, de Luca C, Schenk S, Lu JC, Bandyopadhyay G, Verma IM, Olefsky JM (2009). Hematopoietic cell-specific deletion of Toll-like receptor 4 ameliorates hepatic and adipose tissue insulin resistance in high-fat-fed mice. Cell Metabolism.

[100] Li DY, Yang M, Edwards S, Ye SQ (2013). Nonalcoholic fatty liver disease: for better or worse, blame the gut microbiota?. JPEN. Journal of Parenteral and Enteral Nutrition.

[101] Farrell GC (2005). Signaling links in the liver: knitting SOCS with fat and inflammation. Journal of Hepatology.

[102] Collino M, Aragno M, Castiglia S (2010). Pioglitazone improves lipid and insulin levels in overweight rats on a high cholesterol and fructose diet by decreasing hepatic inflammation. British Journal of Pharmacology.

[103] Emanuelli B, Peraldi P, Filloux C, Chavey C, Freidinger K, Hilton DJ, Hotamisligil GS, Van Obberghen E (2001). SOCS-3 inhibits insulin signaling and is up-regulated in response to tumor necrosis factor-α in the adipose tissue of obese mice. Journal of Biological Chemistry.

[104] Ueki K, Kondo T, Tseng YH, Kahn CR (2004). Central role of suppressors of cytokine signaling proteins in hepatic steatosis, insulin resistance, and the metabolic syndrome in the mouse. PNAS.

[105] Djurhuus MS, Skøtt P, Hother-Nielson O, Klitgaard NA, Beck-Nielsen H (1995). Insulin increases renal magnesium excretion: a possible cause of magnesium depletion in hyperinsulinaemic states. Diabetic Medicine.

[106] Chaudhary DP, Sharma R, Bansal DD (2010). Implications of magnesium deficiency in type 2 diabetes: a review. Biological Trace Element Research.

[107] Kumeda Y, Inaba M (2005). Metabolic syndrome and magnesium. Clinical Calcium.

[108] Steinberg D, Parthasarathy S, Carew TE, Khoo JC, Witztum JL (1989). Beyond cholesterol. Modifications of low-density lipoprotein that increase its atherogenicity. New England Journal of Medicine.

[109] Haberland ME & Steinbrecher UP. Modified low-density lipoprotein: diversity and biological relevance in atherogenesis. In *Molecular Genetics of Coronary Artery Disease: Candidate Genes and Processes in Artherosclerosis. Monographs in Human Genetics*. Eds AJ Lusis, JI Rotter & RS Sparks, vol 14, pp 35–61. Basel, Switzerland: Kargel, 1992.

[110] Böhm I, Heverhagen JT, Behe M, Greschus S, Willinek W, Lohmaier S, Wilhelm K, Block W, Träber F, Schild H (2007). Molecular imaging of apoptosis in cardiovascular diseases. RöFo: Fortschritte auf dem Gebiete der Röntgenstrahlen und der Nuklearmedizin.

[111] Geelen SN, Lemmens AG, Terpstra AH, Wensing T, Beynen AC (2001). High density lipoprotein cholesteryl ester metabolism in the pony, an animal species without plasma cholesteryl ester transfer protein activity: transfer of high density lipoprotein cholesteryl esters to lower density lipoproteins and the effect of the amount of fat in the diet. Comparative Biochemistry and Physiology. Part B, Biochemistry and Molecular Biology.

[112] Johnson PJ, Slight S, Ganjam VK (2002). Glucocorticoids and laminitis in the horse. Veterinary Clinics of North America. Equine Practice.

[113] Pollitt CC (2004). Equine laminitis. Clinical Techniques in Equine Practice.

[114] Knowles EJ, Withers JM, Mair TS (2012). Increased plasma fructosamine concentrations in laminitic horses. Equine Veterinary Journal.

[115] Wyse CA, McNie KA, Tannahill VJ, Prevalence Bailey SR, Habershon-Butcher JL, Ransom KJ (2008). Hypertension and insulin resistance in a mixed-breed population of ponies predisposed to laminitis. American Journal of Veterinary Research.

[116] De Laat MA, Sillence MN, McGowan CM, Pollitt CC (2012). Continuous intravenous infusion of glucose induces endogenous hyperinsulinaemia and lamellar histopathology in Standardbred horses. Veterinary Journal.

[117] Cusi K, Maezono K, Osman A (2000). Insulin resistance differentially affects the PI 3-kinase- and MAP kinase-mediated signaling in human muscle. Journal of Clinical Investigation.

[118] Leclercq IA, Da Silva Morais A, Schroyen B (2007). Insulin resistance in hepatocytes and sinusoidal liver cells: mechanisms and consequences. Journal of Hepatology.

[119] Mancia G, Bousquet P, Elghozi JL (2007). The sympathetic nervous system and the metabolic syndrome. Journal of Hypertension.

[120] Muniyappa R, Montagnani M, Koh KK (2007). Cardiovascular actions of insulin. Endocrine Reviews.

[121] Muniyappa R, Iantorno M, Quon MJ (2008). An integrated view of insulin resistance and endothelial dysfunction. Endocrinology and Metabolism Clinics of North America.

[122] Gauff FC, Patan-Zugaj B, Licka TF (2014). Effect of short-term hyperinsulinemia on the localization and expression of endothelin receptors A and B in lamellar tissue of the forelimbs of horses. American Journal of Veterinary Research.

[123] Lüscher TF, Barton M (2000). Endothelins and endothelin receptor antagonists: therapeutic considerations for a novel class of cardiovascular drugs. Circulation.

[124] Shiffrin EL (2001). Role of endothelin-1 in hypertension and vascular disease. American Journal of Hypertension.

[125] Mazzuca MQ, Khalil RA (2012). Vascular endothelin receptor type B: structure, function and dysregulation in vascular disease. Biochemical Pharmacology.

[126] De Laat MA, Pollitt CC, Kyaw-Tanner MT, McGowan CM, Sillence MN (2013). A potential role for lamellar insulin-like growth factor-1 receptor in the pathogenesis of hyperinsulinaemic laminitis. Veterinary Journal.

[127] Laviola L, Natalicchio A, Giorgino F (2007). The IGF-I signaling pathway. Current Pharmaceutical Design.

[128] Tsujimoto H, Ono S, Efron PA, Scumpia PO, Moldawer LL, Mochizuki H (2008). Role of Toll-like receptors in the development of sepsis. Shock.

[129] Belknap JK, Mooreb JN, Crouserc EC (2009). Sepsis – from human organ failure to laminar failure. Veterinary Immunology and Immunopathology.

